# DNA methyltransferase 3 beta mediates the methylation of the microRNA-34a promoter and enhances chondrocyte viability in osteoarthritis

**DOI:** 10.1080/21655979.2021.2005308

**Published:** 2021-11-29

**Authors:** Shouliang Xiong, Yong Zhao, Tiantong Xu

**Affiliations:** a Department of Orthopedics, The First Affiliated Hospital of Wannan Medical College, Wuhu, Anhui, P.R. China; b Department of Orthopedics, The Central Hospital of Fengxian District, Shanghai, P.R. China; c Department of Spine Surgery, Tianjin Union Medical Center, Tianjin, P.R. China

**Keywords:** DNMT3B, microRNA-34a, MCL1, osteoarthritis, PI3K/AKT pathway

## Abstract

Osteoarthritis (OA) is characterized by destruction of articular cartilage with an imbalance between synthesis and degradation of extracellular matrix (ECM). In the current study, we explored the role of microRNA-34a (miR-34a) and the behind epigenetic mechanism in the degradation of ECM in OA. Using miRNA-based microarray analysis, we found that miR-34a was overexpressed in cartilage tissues of OA patients relative to patients with acute traumatic amputations. Moreover, its expression was positively correlated with the ECM degradation and inflammation. Mechanistically, miR-34a targeted MCL1, and possible target genes of miR-34a were enriched in the PI3K/AKT pathway. Furthermore, DNMT3B inhibited miR-34a by promoting miR-34a methylation. Functional experiments using CCK-8, flow cytometry, Safranin O staining, RT-qPCR, ELISA, Western blot, and HE staining revealed that miR-34a inhibitor suppressed ECM degradation and inflammatory response of chondrocytes and cartilage tissues. By contrast, downregulation of DNMT3B and MCL1 reversed the repressive effects of miR-34a inhibitor *in vitro* and *in vivo*. Altogether, our findings establish that silencing of miR-34a by DNMT3B could effectively reduce chondrocyte ECM degradation and inflammatory response in mice by targeting MCL1 and mediating the downstream PI3K/AKT pathway. This present study revealed that miR-34a knockdown might develop a novel intervention for OA treatment.

## Introduction

Over 10% of the world population display clinical symptoms of osteoarthritis (OA), distressing most individuals above the age of 65 and causing substantial economic and social burden [1]. In OA, chondrocytes and other cells in the synovium and joint tissues are activated as a result of the exposure to abnormal environmental insults, including mechanical stress, inflammatory cytokines, or altered volumes of matrix proteins [2]. During the onset of OA, the cartilage extracellular matrix (ECM) undergoes remodeling and the loss of flexibility, and lesions of the ECM directly contribute to abnormal behavior of chondrocytes, embedded in the articular cartilage, which further aggravates the disease [3]. The molecular mechanisms resulting in an impaired matrix turnover have not been fully elucidated, however cellular senescence, increased expression of inflammatory mediators as well as oxidative stress are all important drivers of the OA development [4]. Therefore, the effective prevention of ECM degradation and the ensuing inflammatory response might become one of the therapeutic strategies for OA treatment.

MicroRNAs (miRNAs) are small double-stranded RNAs, which regulate gene expression negatively and have been evidenced to have major roles during chondrocyte development and cartilage homeostasis [5]. In the pathogenesis of OA, miRNAs have the biological functions of modulating chondrocyte apoptosis and proliferation, ECM metabolism, as well as inflammatory response [6]. MiR-34a is a highly conserved miRNA in many species, and miR-34a has been implicated to serve as a tumor suppressor by targeting many oncogenes associated with cell proliferation, apoptosis and invasion [7]. Interestingly, miR-34a-5p expression was observed to be drastically promoted in plasma, cartilage and synovium of OA patients at late stages and in cartilage and synovium of mice treated with destabilization of the medial meniscus [8]. More importantly, injection of lentiviral vectors containing anti-miR-34a sequence intra-articularly ameliorated the OA symptoms in rats [9]. Therefore, the importance of miR-34a has been preliminarily determined in OA. However, its specific role in regulating ECM degradation and inflammatory response needs to be further explored. Furthermore, DNA methylation is one of the most important epigenetic mechanisms in remediating gene expression, which is strongly involved in aging-bone diseases, such as OA [10,11]. DNA methylation are orchestrated by a family of DNA methyltransferase (DNMTs), including DNMT1, DNMT3a and DNMT3B, and the loss of DNMT3B resulted in the onset of OA-like conditions, such as cartilage matrix loss and premature chondrocyte hypertrophy in mice, which hinted that DNMT3B functioned as an OA suppressor [12]. Hypermethylation of CpG sites in the miR-29b promoter region caused by DNMT3B led to a decline in miR-29b expression in OA chondrocytes [13]. Therefore, we postulated that the expression of miR-34a could also be controlled by DNMT3B in OA chondrocytes. In addition, the importance of the PI3K/AKT pathway has been highlighted in the cartilage degradation and chondrocyte activities in OA [14,15]. The goal of this study is to assess the effect of miR-34a, regulated by DNMT3B, on ECM degradation and inflammatory response in chondrocytes and a rat model with OA by regulating the PI3K/AKT pathway.

## Materials and methods

### Samples

OA cartilage specimens were harvested from 55 patients (mean age 58.16) who underwent arthroscopic joint debridement or knee debridements from January 2015 to January 2019. The status of articular cartilage regeneration was assessed by International Cartilage Repair Society macroscopic score as follows: grade I, superficial lesions, fissures and cracks, soft indentations; grade II, abrasion and depth of cartilage defects less than 50%; Grade III, damage more than half of the cartilage thickness but not reaching the subchondral bone; Grade IV, full-thickness tear combined with subchondral bone exposure. Normal cartilage specimens were acquired from 31 patients (mean age 57.63) with acute traumatic amputations. The clinicopathological characteristics of the participants are shown in Table 1. Patients with rheumatoid arthritis and septic arthritis were excluded from our study. The whole cartilage and a few subchondral bones of the femoral condyles were clinically excised from all participants, and then the samples were immediately placed in liquid nitrogen and cryopreserved. Our research obtained approval of the ethics committee of the First Affiliated Hospital of Wannan Medical College (Approval number: LLSC-2014-025). Fully informed written consent was acquired from every participant.

**Table 1.** Clinicopathological characteristics of participants

Clinicopathological characteristics	Normal (n = 31)	OA (n = 55)
Age		
<**60**	20	32
≥ **60**	11	23
Gender		
Male	14	30
Female	17	25
ICRS score		
I stage	NA	15
II stage	NA	26
III **stage**	NA	12
IV **stage**	NA	2

OA, osteoarthritis; ECM, extracellular matrix; ICRS, International Cartilage Repair Society; NA, not applicable.

### miRNA microarray analysis

According to the stratified sampling method, the OA cartilage tissues of three patients were randomly selected based on age (45, 58, 72 years old, respectively) for miRNA microarray analysis with the normal tissues from controls of the same age. Probes were resuscitated in 50 mM SpotArray 24 Microarray Printing System (PerkinElmer Life Sciences Inc., Boston, MA, USA). RNA was collected using the TRIzol method (Thermo Fisher Scientific Inc., Waltham, MA, USA). In brief, the tissues were first cut into small pieces and placed in a homogenizer, and 1 mL of Trizol was added to 50 mg of tissue for homogenization. The sample was left at room temperature for 5 min to allow sufficient lysis, and 0.2 mL of chloroform was mixed with 1 mL of Trizol for 15 s and left at room temperature for 3 min. After being centrifuged at 12,000 g for 15 min at 4°C, the upper colorless aqueous phase containing the total RNA was aspirated into a new centrifuge tube, mixed well with 0.5 mL isopropanol and precipitated overnight at −70°C. After another centrifugation at 12,000 g for 10 min at 4°C, the RNA precipitate was observed at the bottom of the tube, followed by another centrifugation at 7500 g for 5 min at 4°C with 1 mL 75% ethanol. The supernatant was discarded, and the RNA was obtained. The RNA was denatured at 65°C for 5 min, and the reverse transcription reactions were implemented using random primers and avian myelocytoma virus reverse transcriptase (Thermo Fisher). A solution containing 100 ng RNA or more served as a template, which was added with 1 μL specific miRNA reverse transcription primer and made up to 12 μL with ribonuclease-free deionized water. After the addition of 4 μL of 5 × ES Reaction Mix, 2 μL 10 mM dNTPs, 1 μL RNA inhibitor and 1 μL reverse transcriptase in sequence, complementary DNA (cDNA) was amplified on a PCR instrument (Thermo Fisher) at 25°C for 10 min, at 40°C for 60 min, and at 70°C for 10 min. miRNAs were labeled with Cy3. PCR products were supplemented to hybridization buffer (Ambion, Austin, TX, USA), and the solution was hybridized with the probes overnight at 42°C and then scanned using a ScanArray Express Microarray Scanner (PerkinElmer). Scanned data were analyzed using ScanArray Express version 1.0. Spots with expression below 300 were excluded, and miRNAs with at least 2-folds change in expression were selected for the heatmap plotting.

### RT-qPCR

Intracellular RNA was isolated from chondrocytes and tissues using the RNeasy Plus Mini kit (74,134, Qiagen, Hilden, Germany). The collected fresh cartilage tissues were grounded in liquid nitrogen, while the cells were directly detached and centrifuged. The samples were centrifuged for 3 min at 12,000 g with lysis buffer and mixed with 70% ethanol. A 700 μL sample containing precipitate was added to the RNeasy spin column and centrifuged at 8000 g for 15 s. The effluent was discarded. After another 15-s centrifugation at 8000 g with 700 μL Buffer RW1, the samples were centrifuged with 500 μL Buffer RPE for two times. The precipitate was washed with 50 μL RNase-free water to obtain RNA. cDNA was acquired by reverse transcription of total RNA using M-MLV reverse transcriptase (Promega Corporation, Madison, WI, USA). The reverse transcription primer used for DNMT3B and myeloid cell leukemia 1 (MCL1) detection was oligo(dT), and the reverse transcription primer for miR-34a expression assessment was designed on the basis of the universal stem-loop primer method. Expression of DNMT3B, MCL1, and miR-34a was evaluated using SYBR Green qPCR SuperMix (Thermo Fisher scientific). The relative quantity of gene expression was calculated with 2^−ΔΔCT^ method, and expression of all genes was reflected by Foldchange values. The small nuclear RNA U6 and glyceraldehyde-3-phosphate dehydrogenase (GAPDH) served as endogenous controls. Primer sequences are available in Table 2.

**Table 2.** Primers used for RT-qPCR

Gene name	Sequence **(5ʹ-3ʹ)**
miR-34a-F	ACACTCCAGCTGGGTGGCAGTGTCTTAGCTGGT
miR-34a-R	CTCAACTGGTGTCGTGGA
U6-F	CTCGCTTCGGCAGCACA
U6-R	AACGCTTCACGAATTTGCGT
DNMT3B-F	AGGGAAGACTCGATCCTCGTC
DNMT3B-R	GTGTGTAGCTTAGCAGACTGG
MCL1-F	AAGAGGCTGGGATGGGTTTGTG
MCL1-R	TGGTGGTGGTGGTGGTTGG
MMP3-F	CTGGACTCCGACACTCTGGA
MMP3-R	CAGGAAAGGTTCTGAAGTGACC
MMP13-F	TTGCAGAGCGCTACCTGAGATCAT
MMP13-R	TT TGCCAGTCACCTCTA AGCCGAA
COL II-F	GGTGGAGCAGCAAGAGCAA
COL II-R	AGTGGACAGTAGACGGAGGAAA
iNOS-F	CTGGCAAGCCCAAGGTCTAT
iNOS-R	GGAGGCTCCGATCAATCCAG
COX-2-F	ATCATAAGCGAGGGCCAGCT
COX-2-R	AAGGCGCAGTTTACGCTGTC
TNF-α-F	CATCCGTTCTCTACCCAGCC
TNF-α-R	AATTCTGAGCCCGGAGTTGG
IL-6-F	CCCACCCTCCAACAAAGATT
IL-6-R	GCTCCAGAGCAGAATGAGCTA
GAPDH-F	TGGTCACCAGGGCTGCTT
GAPDH-R	AGCTTCCCGTTCTCAGCC

RT-qPCR, reverse transcription-quantitative polymerase chain reaction; F, forward; R, reverse; miR-34a, microRNA-34a; DNMT3B, DNA methyltransferase 3 beta; MMP, matrix metallopeptidase; COL II, type II collagen; iNOS, inducible nitric oxide synthase; COX-2, cyclooxygenase-2; TNF-α, tumor necrosis factor alpha; IL-6, interleukin-6; GAPDH, glyceraldehyde-3-phosphate dehydrogenase.

### In situ hybridization

Expression of miR-34a in collected OA tissues and normal controls was assessed by *in situ* hybridization. Paraffin-embedded sections were dewaxed in xylene and rehydrated in an ethanol gradient (anhydrous ethanol, 95%, 90%, 85%, 80%, 75% for 10 min respectively). The tissues were incubated overnight at 42°C with biotin-labeled locked nucleic acids (LNA) scrambled control probes or LNA miR-34a antisense probes (Exiqon, Denmark). After thorough washing in sodium chloride-sodium citrate (SCC) buffer gradient diluted with phosphate-buffered saline (PBS)/Tween (4× SSC, 2× SSC, 1× SSC, and 0.5× SSC), the sections were incubated with 0.02 mol/L streptavidin-biotin-alkaline phosphatase complex (Wuhan Boster Biological Technology Co., Ltd., Wuhan, Hubei, China) for 1 h at 42°C and treated with 10 mg/mL 5-Bromo-4-chloro-3-indolyl phosphate (Boster) for 0.5 h at room temperature. miR-34a-positive cells were purple in color. The sections were stained with eosin before sealing to show the histological structure and observed under an inverted microscope (Carl Zeiss, Oberkochen, Germany) for evaluation.

### Isolation, primary culture and transfection of human cartilage chondrocytes

After removing the fibrous connective tissues under aseptic conditions, the clinically collected cartilage tissues were sectioned into 1-mm^3^ sizes. The tissues were washed with PBS containing penicillin and gentamicin and subjected to sequential detachment with 0.25% trypsin (at 1:5) for 0.5 h and with 0.2% collagenase Type II (at a ratio of 1:5) for 16 h. After centrifugation, the cell suspension was filtered through a 200-mesh sieve filter at 1000 rpm for 5 min. The supernatant was discarded, and the cells were plated at 1 × 10^5^ cells/mL in complete medium (Thermo Fisher scientific) containing 10% fetal bovine serum (FBS) and incubated at 37°C in 5% CO_2_. The cells were subjected to passage (P), and the primary cells were P1. The chondrocytes at P2 to P4 were selected for the following experiment.

We inserted 50 pmol miR-34a inhibitor (miR20004557-1-5, Ribobio, Guangzhou, Guangdong, China) and 50 pmol small interfering RNA (siRNA) targeting DNMT3B (si-DNMT3B), si-MCL1 (Applied Biosystems, Table 3) and their respective negative controls (miR-34a control or miR-34a in + NC) into pEGP-miR (Cell Biolabs, San Diego, CA, USA) and PCMV6-XL5 (OriGene Technologies, Beijing, China). The gene fragment was sequenced correctly and cut off with restriction endonuclease, and the vector was digested. The linearized vector and fragment were ligated overnight at 4°C. Lipofectamine 2000 reagent (11668030, Thermo Fisher scientific) was used to perform transfection. Denuclease water (400 μL) was added to the tube and shaken for 10 s, and the mixture was prepared with liposome and plasmids (2:1) to transfect the cells. In short, 4 μL plasmid was added with 2 mL serum-free medium, followed by the addition of 8 μL Lipofectamine 2000. The mixture was left at room temperature for 15 min. The cells were added in the mixture and placed back in the incubator for 6 h, after which the complete medium was added for a 48-h culture for subsequent experiments.

**Table 3.** Sequence used for transfection

Target	Sequence **(5ʹ-3ʹ)**
si-DNMT3B	Guide: UUUACUUGGGCCACUUAACCC
	Passenger: GUUAAGUGGCCCAAGUAAACC
si-MCL1	Guide: UCAACUAUUGCACUUACAGUA
	Passenger: CUGUAAGUGCAAUAGUUGACU

Si, small interfering RNA; DNMT3B, DNA methyltransferase 3 beta; MCL1, myeloid cell leukemia 1.

### Bioinformatics analysis

Bioinformatics analysis was performed by R (Version 3.6.3, NIH, Bethesda, MD, USA), and relevant graphs were drawn. Multifactor correlation analysis was performed by corrgram package (R, NIH), and a correlation heatmap was drawn. Gene Ontology (GO) enrichment and KEGG enrichment analyses were performed by ClusterProfiler package (Bioconductor, Seattle, WA, USA) and visualized by Barplot package (version 3.6.3, R). GO annotation information and KEGG pathway data were downloaded from GO database (http://www.bioconductor.org/packages/release/data/annotation/) and KEGG database (https://www.kegg.jp/kegg/rest/keggapi.html), respectively. The CpG Island of miR-34a promoter was obtained by MethPrimer (http://www.urogene.org/cgi-bin/methprimer).

### Luciferase reporter gene assay

As suggested by the StarBase website (http://starbase.sysu.edu.cn/), MCL1 is one of the potential targets for miR-34a. The corresponding sequences contained the wild-type (WT) sequence of miR-34a targeting the MCL1 3ʹUTR and the mutant (MT) (Sangon, Shanghai, China). The gene sequence and the plasmid were cleaved by restriction endonuclease, and the T4 DNA ligase was incubated overnight at 4°C with the target fragment and the pMIR-reporter reporter plasmid. The sequences were subcloned into the PCMV6 vector (Promega) to construct the corresponding MCL1-WT plasmid and MCL1-MT plasmid. The cells were co-transfected as follows: pEGP-miR-34a inhibitor + MCL1-WT, pEGP-miR-34a inhibitor + MCL1-MT, pEGP-miR-34a control + MCL1-MT and pEGP-miR-34a control + MCL1-WT. Lipofectamine 2000 reagent (11668030, Thermo Fisher scientific) was used to perform the above transfection. After 24 h, the luciferase activity was assessed using a dual-luciferase assay kit (E1910, Promega) as per the manufacturer’s instructions. The cells were incubated for 3 min by adding lysis solution, and firefly luciferase activity was detected by adding 1 mL Buffer, after which the reaction was terminated by adding termination solution. Then, 1 mL assay Substrate was added to detect Renilla luciferase activity, relative luciferase activity = Renilla luciferase activity/firefly luciferase activity.

### Quantitative methylation specific PCR (qMSP)

DNA sample (1 μg) was added into a 1.5-mL eppendorf tube containing 50 μL water and supplemented with 5.5 μL 2 mol/L NaOH and incubated for 10 min. After the addition of 3 μL 10 mmol/L hydroquinone, 520 μL 3 mol/L sodium bisulfite and enough mineral oil (about 50 μL) to cover the aqueous phase, the samples were cultured for another 16 h at 50°C. After the oil layer was removed, 1 mL DNA Wizard reagent (Promega) was added. The mixture was added to the elution column, eluted with isopropanol and incubated with 50 μL of 60°C water and 3 mol/L NaOH for 5 min at room temperature. Then, the mixture was added with 1 μL 10 mg/mL glycoside ligand and 17 μL 10 mol/L amyl acetate and chilled ethanol for an overnight precipitation at −20°C and centrifuged for 20 min to remove the supernatant. After washing with ethanol and dissolving with 20 μL water, the PCR reaction was carried out using Methylamp Universal Methylated DNA Kit (P-1011-2, AmyJet Scientific Inc., Wuhan, Hubei, China). Methylated human genomic DNA (5 µL) was added to the working solution and incubated at 65°C for 90 min, followed by centrifugation at 12,000 g for 20 s. The modified DNA was eluted by adding 200 µL 70% ethanol to the column and centrifuging at 8000 g for 20 s. The DNA was subjected to PCR reaction to detect the methylation modification.

### Chromatin immunoprecipitation (ChIP)

The binding relationship between DNMT3B and miR-34a was analyzed using an EZ-ChIP kit (#17-371, Millipore Corp, Billerica, MA, USA). The cells (1 × 10^5^) were cross-linked in 1% formaldehyde for 10 min and lysed in sodium dodecyl sulfate (SDS) lysis buffer containing 40 mM Tris-Acetate, 1 mM ethylenediaminetetraacetic acid, 20 mM sodium acetate, 1% SDS, followed by sonication to shear DNA. Lysates diluted with ChIP dilution buffer were immunoprecipitated overnight at 4°C with antibodies to IgG (1:300, ab99757, Abcam, Cambridge, UK) and DNMT3B (1:100, sc-81252. Santa Cruz Biotechnology Inc., Santa Cruz, CA, USA). Antibody-chromatin complexes were precipitated with 60 µL protein G agarose at 4°C for 1 h, then washed and eluted. The DNA was de-crosslinked, purified, and analyzed by real-time PCR as above mentioned.

### Cell counting kit-8 (CCK-8)

Primarily cultured chondrocytes transfected with miR-34a inhibitor, miR-34a in + si-DNMT3B, miR-34a in + si-MCL1 and their respective negative controls (miR-34a control or miR-34a in + NC) were cultured in 96-well cell culture plates with 5 × 10^3^ cells in each well. After 72 h of incubation, CCK-8 solution (Thermo Fisher scientific) was added to the medium at a ratio of 10 mL/100 mL for another 4-h incubation at 37°C. Optical density (OD) was detected at 450 nm using an iMark microplate reader (Bio-Rad Laboratories, Hercules, CA, USA), and cell viability was calculated from the OD values of the reference standard curve.

### Flow cytometry

The cells transfected with oligonucleotides were trypsinized and fixed in ice-cold 70% ethanol for 30 min. Afterward, the 5 × 10^6^ cells were incubated with 20 mg/mL RNase (Sigma-Aldrich Chemical Company, St Louis, MO, USA) at 37°C for 60 min. For apoptosis analysis, the cells were stained with fluorescein isothiocyanate-Annexin V and propidium iodide (Beyotime, Beijing, China) for 5 min, respectively. The apoptotic cells were detected using a FACSCalibur flow cytometer (BD Biosciences, San Jose, CA, USA).

### The animal model of OA

Experiments were conducted as per the protocol approved by the Institutional Review Board of The First Affiliated Hospital of Wannan Medical College (Approval number: 20190127), and the study was implemented in compliance with the Institutional Animal Care Standards. A total of 35 Sprague-Dawley rats (2-month-old, Beijing Vital River Laboratory Animal Technology Co., Ltd., Beijing, China) were housed in a specific pathogen-free facility and kept under controlled temperature and humidity on a 12/12 h light/dark cycle with ad libitum access to chow and drinking water.

The rats were divided into 7 groups (n = 5), and except for the normal group, the remaining 30 rats were modeled with anterior cruciate ligament transection and medial meniscus resection. In brief, 1% pentobarbital sodium (40 mg/kg) was administered intraperitoneally once for anesthesia, and a routine medial paraprosthetic incision was made on the right posterior knee. The skin was cut, and the subcutaneous tissues were carefully separated. The P-bone was dislocated laterally, and the joint capsule was cut to expose the knee joint. The anterior cruciate ligament of the knee joint was seen in flexion and cut with scissors. The joint capsule and skin were sutured. Penicillin (200,000 U) was administered intramuscularly on the first postoperative day. For the rats in the normal group, identical procedures were performed while keeping the ligaments and medial menisci intact, followed by direct suturing and disinfection. During the experiment, rats were monitored daily for behavior and health.

The miR-34a control, miR-34a inhibitor, miR-34a in + NC, miR-34a in + si-DNMT3B and miR-34a in + si-MCL1 cloned into recombinant lentiviral vectors were obtained from Genechem (Shanghai, China). Starting from the first week after surgery, 100 μL saline plus 1 × 10^9^ PFU lentiviral vector was injected intra-articularly into OA rats every 7 days. At the 72nd day of treatment, the rats were euthanized by an intraperitoneal injection of 1% pentobarbital sodium at 150 mg/kg. Synovial fluid and intact joints were collected from rats for subsequent experiments.

### Safranin O staining

The intact joints of rats were removed and fixed in 4% paraformaldehyde, and the tissues were routinely paraffin-embedded and sectioned at 5-μm thickness using a microtome. Paraffin-embedded sections containing intact joint sections were dewaxed, hydrated, stained with hematoxylin for 10 s, then rinsed with water for a few min, stained with 0.1% fast green aqueous solution for 5 min, immersed in glacial acetic acid for 5 s, and dried. After incubation in 0.5% Safranin O solution (Takara Holdings Inc., Kyoto, Japan) for 5 min, the sections were dried at room temperature, treated with xylene for 5 min, and finally sealed with a neutral gel (Millipore). The extent of tissue damage was observed under a light microscope (Zeiss). The Mankin score was used to define the extent of histological changes in the samples [16].

### Western blot

The cells (5 × 10^6^) were lysed on ice-cold radio immunoprecipitation assay buffer (Beijing Solabio Life Sciences Co., Ltd., Beijing, China) 48 h after transfection to extract total protein. A total of 30 μg protein were separated by SDS-polyacrylamide gel electrophoresis (separating gel concentration 8%, stacking gel concentration 5%), and protein concentrations were quantified using a bicinchoninic acid assay kit (P0914, Sigma-Aldrich). The protein extracts were transferred onto polyvinylidene fluoride membranes. After being sealed with 5% skimmed milk dissolved in Tris-buffered saline with Tween for 1 h, the membranes were probed with primary antibodies against DNMT3B (1:1000, ab2851, Abcam), MCL1 (1:1200, sc-12756, Santa Cruz Biotechnology), MMP3 (1:2,000, ab52915, Abcam), MMP13 (1:1,500, 701287, Thermo Fisher scientific), COL II (1:1,500, ab34712, Abcam), total-PI3K (1:2,000, ab32089, Abcam), p-PI3K (1:1,000, ab278545, Abcam), total-AKT (1:1,000, ab8805, Abcam), p-AKT(1:1,500, 44–621 G, Thermo Fisher scientific) and GAPDH (1:1,000, G9545, Millipore) overnight at 4°C. After that, the immobilized primary antibodies were detected using a secondary antibody (1:3,000, ab205718, Abcam) at room temperature for 60 min and were visualized with an enhanced chemiluminescence kit (GE Healthcare, Pittsburgh, PA, USA).

### Toluidine blue staining

*In vitro* cartilage-like tissue formation was studied in high-density sediment cultures. A total of 5 × 10^5^ cells were seeded in 0.5 mL Dulbecco’s modified Eagle’s medium containing gentamicin (50 mg/mL), L-glutamine (300 mg/mL), amphotericin B (2.5 mg/mL), ascorbic acid (50 μg/mL) and 0.1% FBS and centrifuged at 500 g for 5 min. The cells were cultured in 15 mL polypropylene tubes for 3 weeks, and the medium was renewed two times a week. The cells were stained with toluidine blue (Sigma-Aldrich) for 10 min and viewed under a stereomicroscope (MVX-10 MacroView Systems, Olympus, Tokyo, Japan) equipped with a DP71 camera (Olympus).

### ELISA

Sterile saline (0.2 mL) was injected into the knee joint of one hind limb of OA and normal rats to extract the synovial fluid. The synovial fluid of rats was placed at 4°C for 30 min, centrifuged at 300 g and then collected for the following experiments. The concentration of inflammatory factors in synovial fluid was measured according to a TNF-α ELISA Kit (E-EL-R2856c, Elabscience Biotechnology Co., Ltd., Wuhan, Hubei, China) and a IL-6 ELISA Kit (E-EL-R0015c, Elabscience). A total of 100 μL standard working solution or sample was added to the corresponding wells for a 1.5-h incubation at 37°C. After that, the samples were incubated for 1 h with 100 μL biotinylated anti-TNF-α or IL-6 working solution, for 30 min with 100 μL horseradish peroxidase conjugated working solution, and for 15 min with 90 μL substrate solution (all at 37°C). Finally, 50 μL termination solution (2 mol/L H_2_SO_4_ solution) was added, and the plate was read immediately at 450 nm using an iMark microplate reader (Bio-Rad). The standard linear equation was plotted, and the inflammatory factor concentration was measured according to the corresponding results.

### Immunohistochemistry

The extracted OA tissues from rats and normal cartilage tissues from sham-operated rats were routinely embedded, dewaxed, hydrated, and then rested for 15 min at room temperature with a drop of 3% H_2_O_2_ and for 15 min at room temperature with a drop of normal goat serum sealant. After washing, the tissues were incubated overnight at 4°C with 50 μL primary antibody to iNOS (1:500, ab115819, Abcam) and COX2 (1:500, ab179800, Abcam) and with secondary antibody (1:500, ab205718, Abcam) for 15 min at 37°C. Horseradish-labeled streptavidin working solution was added dropwise and incubated for 15 min at 37°C for diaminobenzidine color development. After being rinsed with distilled water, the tissues were then counter-stained with hematoxylin for 30 s, dehydrated, and sealed. Cells showing brownish-yellow or brownish-brown granules in the nucleus were considered positive.

### Hematoxylin-eosin (HE) staining

Cartilage tissues were removed from OA and normal rats and fixed in 10% neutral formaldehyde solution for 24 h, followed by dehydration in graded concentrations of ethanol (70%, 80%, 90%, 95% and 100%). The tissues were paraffin-embedded and cut into 5-μm sections after xylene transparency. The sections were stained with hematoxylin (Solarbio) for 4 min, then fractionated in hydrochloric acid ethanol for 10 s. After rinsing, the sections were stained again with eosin (Solarbio) solution for 2 min, fixed with neutral gum, and viewed under a DMM-300D microscope (Shanghai CaiKon Optical Instrument Co., Ltd., Shanghai, China) to observe the cartilage tissue damage and inflammatory cell infiltration.

### Statistics

Three independent experiments were carried out, and the results were expressed as mean ± SD of triplicate values for each experiment. Statistical analyses were carried out by two-tailed unpaired *t* test and one-way or two-way ANOVA, followed by Tukey’s test as appropriate. The level of significance was set at *p* less than 0.05. Statistical tests for data analysis were performed using SPSS 22.0 software (IBM SPSS Statistics, Chicago, IL, USA).

## Results

In this study, we explored the biological role and molecular mechanism of miR-34a in OA. Our data showed that miR-34a was highly expressed in OA. Downregulation of miR-34a alleviated cartilage injury and promoted chondrocyte activity in OA rats. miR-34a, regulated by DNMT3B, mediates OA progression by targeting MCL1. In conclusion, our study showed the role of DNMT3B/miR-34a/MCL1 in OA chondrocyte injury, which meant that targeting these molecules may be a new method for the treatment of OA.

### MiR-34a expression is increased in OA cartilage tissues and chondrocytes

**Figure 1.**
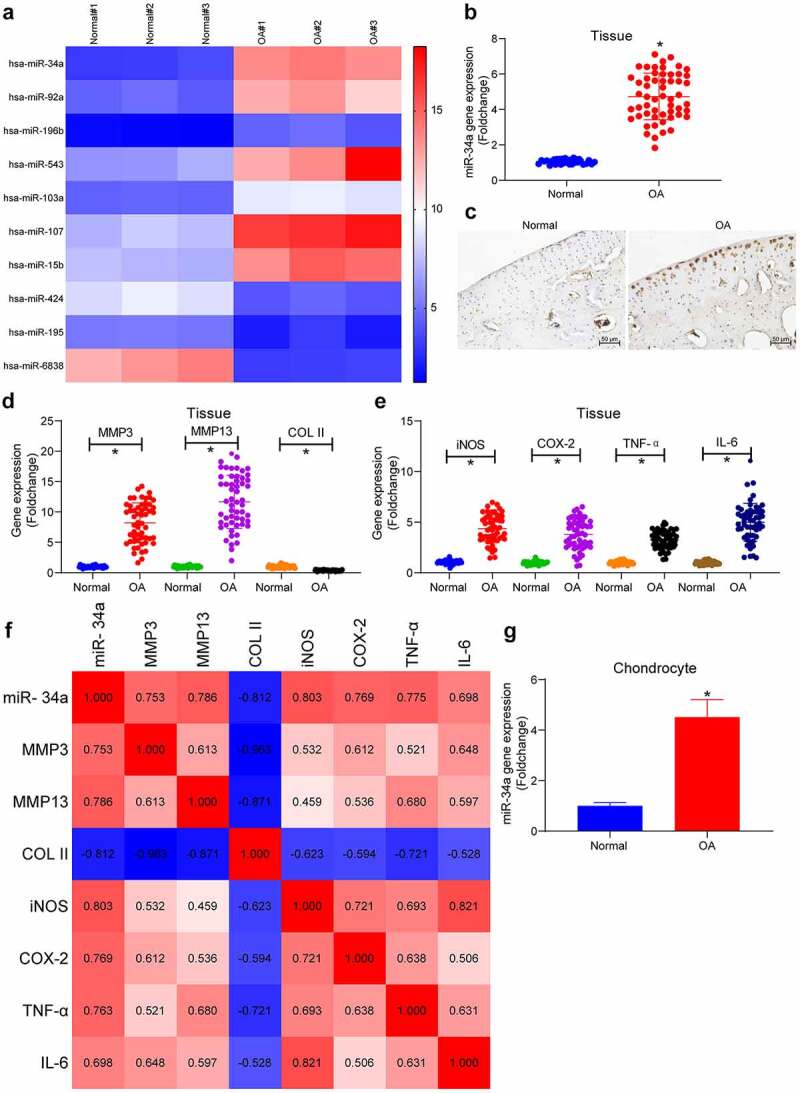
MiR-34a is upregulated in OA cartilage tissues and chondrocytes. (a) Differentially expressed miRNAs in cartilage tissues by miRNA microarray (listed by foldchange), among which 7 were upregulated (miR-34a, miR-92a, miR-196b, miR-543, miR-103a, miR-107, miR-15b), whereas 3 were downregulated (miR-424, miR-195, miR-6838). (b) Detection of miR-34a expression in OA cartilage tissues by RT-qPCR where normal = 31, OA = 55. (c) miR-34a localization and activity in tissues by in situ hybridization. (d) Expression of ECM degradation markers in OA cartilage tissues by RT-qPCR where normal = 31, OA = 55. (e) PCR detection of pro-inflammatory factor expression in OA cartilage tissues where normal = 31, OA = 55. (f) Person’s correlation analysis of correlation between miR-34a expression and ECM degradation markers and inflammatory factors. (g) miR-34a expression in OA chondrocytes by RT-qPCR. Data are expressed as the mean ± SD of three independent experiments. *p < 0.05 compared with normal cartilage tissues or chondrocytes by unpaired *t*-test (panels B and G) or one-way ANOVA (panels D and E)

The miRNA microarray analysis was performed in the collected normal and OA cartilage tissues. As observed in the plotted heatmap of differentially expressed miRNAs in Figure 1a, seven miRNAs were found to be upregulated and three miRNAs were downregulated in OA cartilage tissues, with miR-34a being the most significantly upregulated one (the highest foldchange). Expression of miR-34a was examined by PCR and in situ hybridization. PCR analysis revealed significantly upregulated miR-34a in cartilage tissues of all OA patients (Figure 1b); this was further substantiated by *in situ* hybridization which also revealed that miR-34a was mainly present in chondrocytes and ECM (Figure 1c). In addition, the expression of MMP3 and MMP13 was considerably elevated in OA cartilage tissues compared with normal cartilage tissues, whereas the COL II expression was appreciably downregulated in OA cartilage tissues, suggesting severe degradation of the ECM (Figure 1d). The levels of inflammatory factors in the tissues were also detected, and the pro-inflammatory factors iNOS, COX-2, TNF-α and IL-6 were found to be increased to different degrees, indicating the aggravation of inflammatory response in the cartilage tissue of OA patients (Figure 1e). A correlation heatmap was then plotted where blue boxes denote negative correlations, whereas red boxes refer to positive correlations, and color shades were proportional to the absolute value of correlation coefficients. It was found that miR-34a expression was positively correlated with MMP3, MMP13, iNOS, COX-2, TNF-α and IL-6, while negatively correlated with COL II in cartilage tissues of OA patients (Figure 1f). Thus, miR-34a was positively correlated with the degree of ECM degradation and inflammatory factor levels in OA cartilage tissue. Detection of miR-34a expression in primarily cultured chondrocytes showed a significant upregulation of miR-34a expression in OA chondrocytes relative to normal human chondrocytes (Figure 1g), predicting a critical role of miR-34a in OA cartilage tissue.

### miR-34a targets and inhibits the expression of MCL1

**Figure 2.**
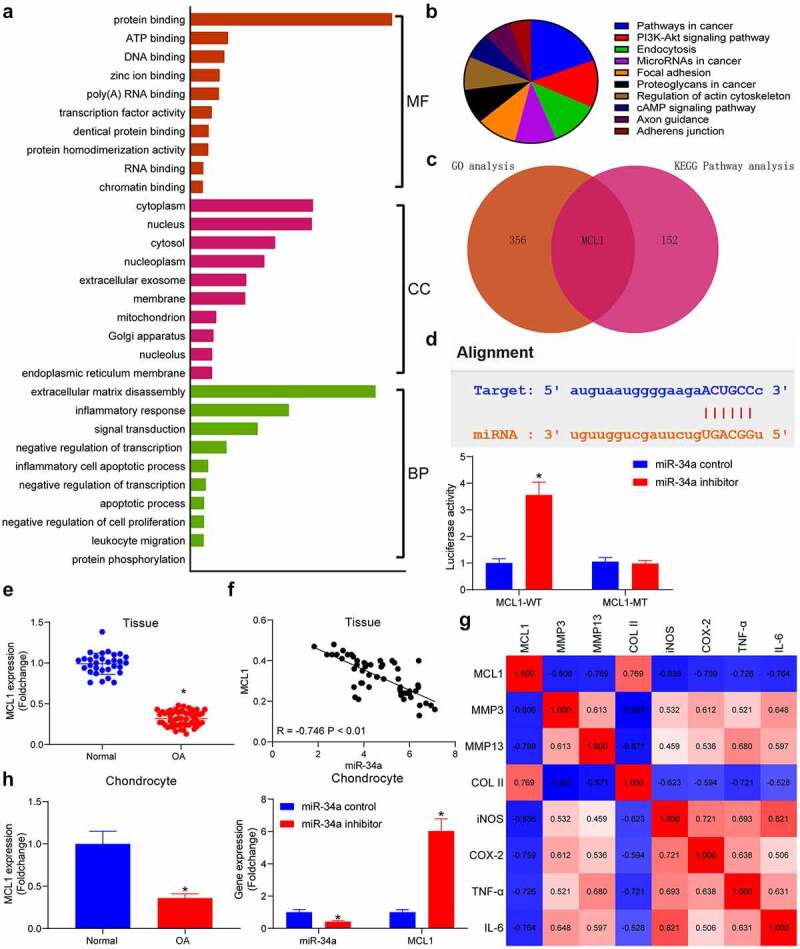
MCL1 is a putative target of miR-34a. (a) Gene Ontology (GO) enrichment analysis of miR-34a target genes. (b) Kyoto Encyclopedia of Genes and Genomes (KEGG) pathway enrichment analysis of miR-34a target genes. (c) Overlapping results of GO analysis and KEGG analysis by Venn analysis. (d) the relationship between miR-34a and MCL1 verified by dual-luciferase assay. (e) Detection of MCL1 expression in cartilage tissues by RT-qPCR where normal = 31, OA = 55. (f) Person’s correlation analysis of correlation between MCL1 and miR-34a in cartilage tissues. (g) Person’s correlation analysis of correlation between MCL1 expression and ECM degradation markers and inflammatory factors. (h) MCL1 expression in chondrocytes with miR-34a inhibitor by RT-qPCR. Data are expressed as the mean ± SD of three independent experiments. *p < 0.05 compared with normal cartilage tissues, chondrocytes or miR-34a control by unpaired *t*-test (panel E) or two-way ANOVA (panels D and H)

To find the downstream mechanism of miR-34a, the putative targets of miR-34a were detected in the Starbase database, which were subjected to enrichment analyses. GO enrichment analysis showed that miR-34a downstream genes were mainly enriched in ECM disassembly, inflammatory response and other processes, and were mainly present in the cytoplasm, performing extracellular matrix degradation in cells (Figure 2a). Meanwhile, KEGG pathway enrichment analysis revealed that genes were mainly enriched in the PI3K/AKT signaling pathway (Figure 2b). During the enrichment analysis, we found that MCL1 was not only a key factor in the PI3K/AKT pathway, but also significantly enriched in ECM degradation and inflammatory response (Figure 2c). As suggested by TargetScan database, the binding site of miR-34a was located in the 3ʹUTR of MCL1. Moreover, the luciferase activity was much higher in the cells transfected with miR-34a inhibitor + MCL1-WT relative to the miR-34a control + MCL1-WT group, while the luciferase activity between cells transfected with the miR-34a inhibitor + MCL1-MT or the miR-34a control + MCL1-MT was not drastically different. These results confirmed that miR-34a had a direct targeting effect on MCL1 (Figure 2d). PCR results displayed that MCL1 was considerably downregulated in OA cartilage tissues (Figure 2e) and conversely correlated with miR-34a expression in OA cartilage tissues (Figure 2f). There were also significant negative correlations between MCL1 expression and the degradation of ECM and the degree of inflammation in OA cartilage tissues (Figure 2g). Assessment of MCL1 content in chondrocytes revealed that MCL1 was significantly downregulated in OA chondrocytes and upregulated in OA chondrocytes with low miR-34a expression (Figure 2h). These results demonstrate the potential inhibitory effects of miR-34a on the expression of MCL1.

### DNMT3B suppresses miR-34a expression through an epigenetic mechanism

**Figure 3.**
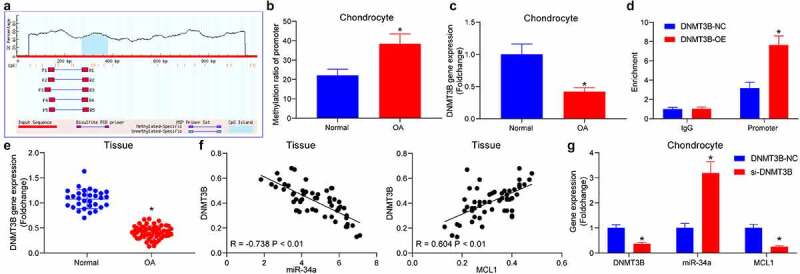
DNMT3B is involved in the upregulation of miR-34a in OA. (a) Bioinformatics analysis of the methylation region of miR-34a. (b) the promoter methylation level of miR-34a in cells examined using qMSP. (c) Detection of DNMT3B expression in OA chondrocytes by RT-qPCR. (d) the binding relationship between DNMT3B and miR-34a verified using ChIP assay. (e) Detection of DNMT3B expression in OA cartilage tissues by RT-qPCR where normal = 31, OA = 55. (f) Person’s correlation analysis of the correlation between DNMT3B and miR-34a expression or MCL1. (g) PCR detection of DNMT3B, miR-34a and MCL1 expression in response to si-DNMT3B. Data are expressed as the mean ± SD of three independent experiments. *p < 0.05 compared with normal cartilage tissues, chondrocytes or DNMT3B-NC by unpaired *t*-test (panels B, C and E) or two-way ANOVA (panels D and G)

To probe the upstream genes of miR-34a, the promoter of miR-34a was obtained, and the CpG Island region was predicted (Figure 3a). The methylation of the miR-34a promoter at −272/380 bp region in cells was analyzed by qMSP, and the methylation of miR-34a was found to be significantly elevated in OA chondrocytes (Figure 3b). To determine the epigenetic regulators of miR-34a in chondrocytes, DNMT3B was revealed to be expressed at a poor level in OA chondrocytes (Figure 3c). Moreover, DNMT3B protein was significantly enriched in the promoter region of miR-34a (Figure 3d). RT-qPCR revealed that DNMT3B was reduced in OA cartilage tissues (Figure 3e) and was negatively correlated with miR-34a and positively correlated with MCL1 expression in OA cartilage tissues (Figure 3f). The expression changes of miR-34a and MCL1 were detected in cells with si-DNMT3B. miR-34a was appreciably elevated after DNMT3B downregulation, while that of MCL1 was drastically reduced (Figure 3g). It is suggested that DNMT3B suppresses miR-34a expression and consequently affects MCL1 expression through an epigenetic mechanism.

### DNMT3B/miR-34a/MCL1 axis regulates the viability of chondrocytes

**Figure 4.**
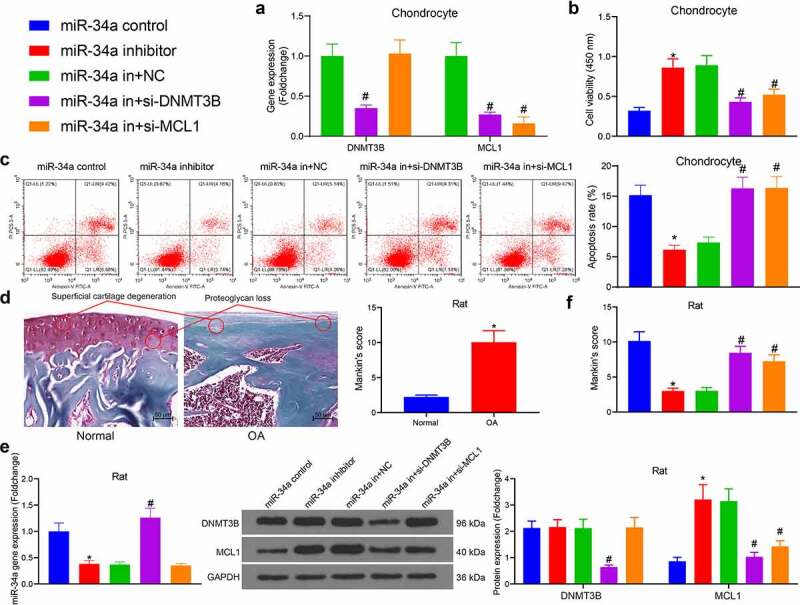
DNMT3B/miR-34a/MCL1 axis regulates the viability of chondrocytes. Chondrocytes or rats (n = 5 for each group) were delivered with miR-34a inhibitor, miR-34a inhibitor + si-DNMT3B, miR-34a inhibitor + si-MCL1 or their respective controls. (a) Expression of DNMT3B and MCL1 in chondrocytes after transfection. (b) CCK8 assay for cell proliferation activity. (c) Detection of apoptotic activity by flow cytometry. (d) Detection of cartilage damage in OA rats *in vivo* by Safranin O staining. (e) miR-34a expression in rat cartilage tissues (left) examined by RT-qPCR and the protein expression of DNMT3B and MCL1 in rat cartilage tissues examined using Western blot (right). (f) Detection of cartilage damage in OA rats by Safranin O staining. Data are expressed as the mean ± SD of three independent experiments. *p < 0.05 compared with miR-34a control, #*p* < 0.05 compared with miR-34a in + NC by unpaired t-test (panel D), one-way (panels B, C and F) or two-way ANOVA (panel E)

The miR-34a inhibitor was administrated into OA chondrocytes, and the expression patterns of DNMT3B and MCL1 were downregulated in cells with reduced miR-34a for rescue experiments, respectively (Figure 4a). CCK-8 assays exhibited that chondrocytes treated with miR-34a inhibitor had significantly increased cell viability compared with the miR-34a control group. Whereas the downregulation of DNMT3B and MCL1 both antagonized the effect of miR-34a inhibitor and significantly decreased cell viability compared with the miR-34a in + NC group (Figure 4b). The effect of aberrant gene expression on cell apoptosis was further assessed using flow cytometry. MiR-34a inhibitor repressed apoptosis in OA chondrocytes, while DNMT3B and MCL1 downregulation reversed this effect to increase cell apoptosis (Figure 4c).

OA rats were then developed and examined for cartilage damage by Safranin O staining. The OA rats showed severe proteoglycan loss and superficial cartilage degeneration after 10 weeks of surgery (Figure 4d). After that, rats developed with OA were further administrated with miR-34a inhibitor, miR-34a in + si-DNMT3B, miR-34a in + si-MCL1 or their respective controls (miR-34a control and miR-34a in + NC). RT-qPCR and Western blot showed that stable downregulation of miR-34a induced MCL1 protein expression, while the downregulation of DNMT3B restored the expression of miR-34a (Figure 4e). Cartilage tissues were collected again for histological assessment. MiR-34a inhibitor in the joint cavity significantly attenuated the cartilage damage induced by surgical resection, while downregulation of DNMT3B and MCL1 reversed the effect of miR-34a inhibitor to worsen cartilage damage, and these results were also validated using the Mankin’s score (Figure 4f). The experiment showed that DNMT3B/miR-34a/MCL1 axis mediates chondrocyte viability *in vitro* and cartilage damage caused by OA *in vivo*.

### DNMT3B/miR-34a/MCL1 axis regulates the degradation of ECM

**Figure 5.**
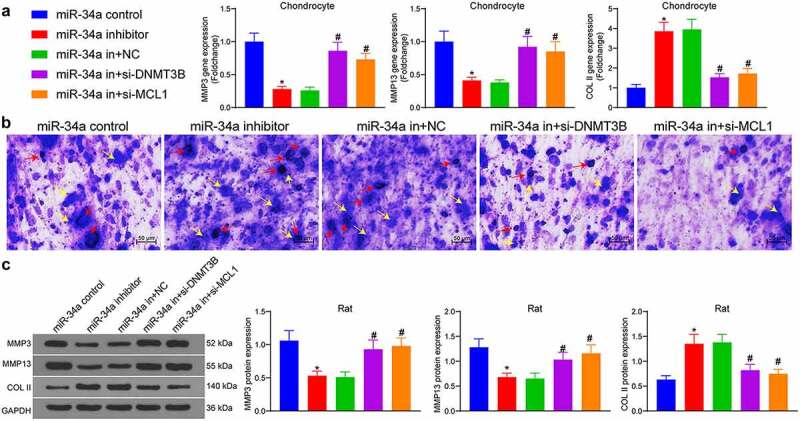
DNMT3B/miR-34a/MCL1 axis regulates the degradation of ECM. (a) Detection of ECM degradation-related markers in chondrocytes by RT-qPCR. (b) ECM degradation examined using Toluidine blue staining where red arrows indicate chondrocytes and yellow arrows indicate ECM. (c) cartilage ECM degradation in rats assessed using Western blot. Data are expressed as the mean ± SD of three independent experiments. *p < 0.05 compared with miR-34a control, #*p* < 0.05 compared with miR-34a in + NC by one-way ANOVA

As indicated by RT-qPCR results, chondrocytes treated with miR-34a inhibitor showed much lower expression of MMP3 and MMP13 and higher expression of COL II. However, miR-34a in + si-DNMT3B and miR-34a in + si-MCL1 significantly promoted expression of MMP3 and MMP13 and lowered that of COL II in cells compared with the miR-34a in + NC group, signifying that ECM degradation was promoted in chondrocytes (Figure 5a). Toluidine blue staining revealed that the chondrocytes were purple-blue in color, and the ECM was blue in color. There were also blue-purple particles in and around the chondrocytes which were secreted and synthesized proteoglycans, showing that the miR-34a inhibitor increased the particles secreted by the cells, and the downregulation of MCL1 and DNMT3B reversed the effect of miR-34a depletion to reduce the proteoglycans secreted by the cells (Figure 5b). The protein expression of ECM markers was examined in rat cartilage tissues as well. The protein expression of MMP3 and MMP13 was downregulated and COL II was induced by miR-34a inhibitor. Conversely, the inhibitory fragment of DNMT3B and MCL1 increased the expression of MMP3 and MMP13 and decreased COL II expression in cartilage tissues of OA rats (Figure 5c). It was thus suggested that DNMT3B/miR-34a/MCL1 axis is involved in the degradation of ECM.

### DNMT3B/miR-34a/MCL1 regulates the inflammatory response in OA

**Figure 6.**
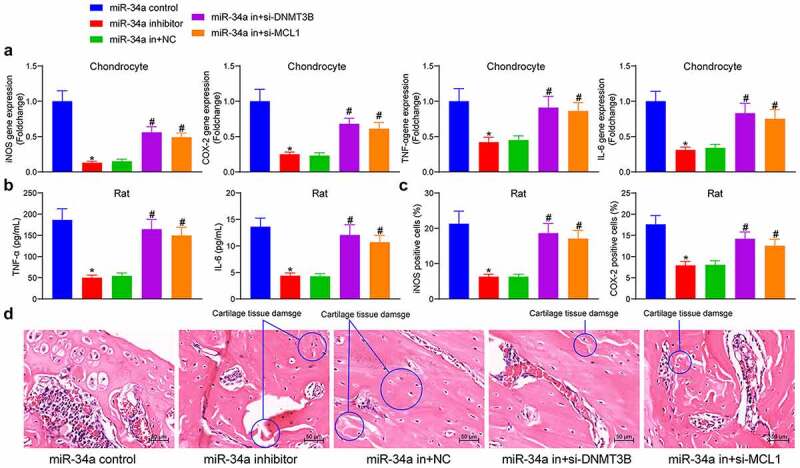
DNMT3B/miR-34a/MCL1 axis regulates the inflammatory response in OA. (a) expression of inflammatory factors in chondrocytes by ELISA. (b) The concentration of TNF-α and IL-6 in synovial fluid of rats measured by ELISA. (c) Relative OD value of iNOS and COX-2 in cartilage tissues examined using immunohistochemical detection. (d) The cartilage tissue damage (circled out) and inflammatory cell infiltration measured using HE staining. Data are expressed as the mean ± SD of three independent experiments. *p < 0.05 compared with miR-34a control, #*p* < 0.05 compared with miR-34a in + NC by one-way ANOVA

The changes in the expression of pro-inflammatory factors were detected in the cells, showing that miR-34a downregulation reduced the expression of iNOS, COX-2, TNF-α and IL-6 in chondrocytes, and depletion of DNMT3B and MCL1 mitigated the effect of miR-34a inhibitor to significantly increase the pro-inflammatory factors in the cells (Figure 6a). To examine the anti-inflammatory effects of miR-34a downregulation in OA animals, we measured the concentrations of TNF-α and IL-6 in the synovial fluid after euthanasia of the animals. Rats delivered with miR-34a inhibitor showed a significant decline in the concentrations of TNF-α and IL-6. However, the concentrations of pro-inflammatory factors TNF-α and IL-6 were significantly augmented after si-DNMT3B or si-MCL1 treatment (Figure 6b). Immunohistochemical staining of inflammation-related proteins showed that the relative OD value of iNOS and COX-2 in rat cartilage tissues was significantly lower in the miR-34a inhibitor group than that in the miR-34a control group, while the treatment of si-DNMT3B and si-MCL1 partially restored the expression of iNOS and COX-2 in cartilage tissues in the presence of miR-34a inhibitor (Figure 6c). HE staining showed that the rats treated with miR-34a inhibitor showed alleviated cartilage degeneration, reduced matrix fissures, increased number of chondrocytes and lessened inflammatory cell infiltration relative to rats with miR-34a control. While the downregulation of MCL1 and DNMT3B inhibited this effect, resulting in increased cartilage tissue damage, reduced cartilage thickness and accentuated inflammatory cell infiltration and fissures in rats (Figure 6d). Reduced inflammatory response in response to miR-34a downregulation and exacerbated inflammatory response following reduced DNMT3B and MCL1 expression were found both in cells and tissues.

### DNMT3B/miR-34a/MCL1 affects chondrocyte viability and OA progression via the PI3K/AKT pathway

**Figure 7.**
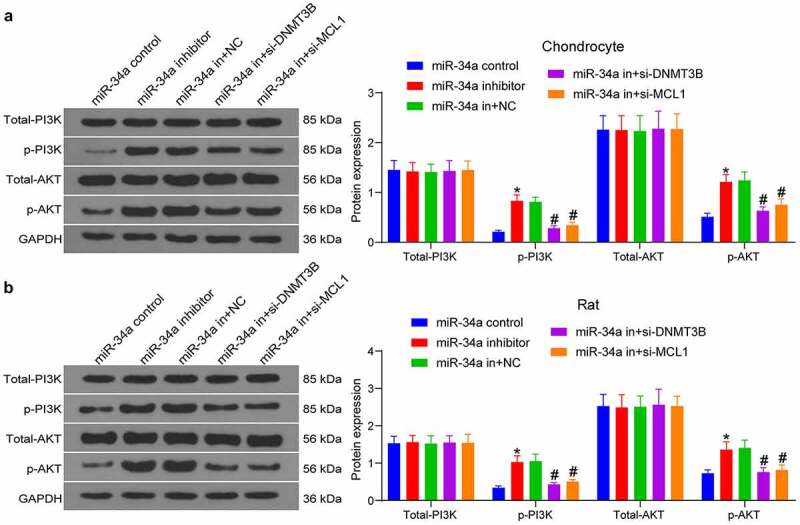
DNMT3B/miR-34a/MCL1 axis governs the activation of the PI3K/AKT pathway in OA. The expression of total and phosphorylated PI3K and AKT in chondrocytes (a) and OA rats (b) measured using Western blot. Data are expressed as the mean ± SD of three independent experiments. *p < 0.05 compared with miR-34a control, #*p* < 0.05 compared with miR-34a in + NC by two-way ANOVA

As predicted in Figure 2b, the PI3K/AKT pathway is the main singling pathway enriched by the target genes of miR-34a. To detect changes in the PI3K/AKT pathway activity after gene expression alteration, we conducted Western blot both in tissues and cells. As expected, miR-34a downregulation increased PI3K/AKT pathway activity, while the silencing of DNMT3B and MCL1 in turn inhibited PI3K/AKT pathway activity in both chondrocytes and rat cartilage tissues (Figure 7a, b). It was suggested that DNMT3B/miR-34a/MCL1 axis affects chondrocytes and OA development through the PI3K/AKT pathway.

## Discussion

Articular cartilage is a kind of tissue whose properties of mechanical support and joint lubrication are dependent on the integrity of ECM which is rich in fibrillar collagens, particularly COL II and large aggregating proteoglycans [17]. A great number of miRNAs found to be engaged in the development of OA via different signaling pathways [18]. COL II is the most abundantly expressed collagen in articular cartilage, which is closely related to OA progression [4]. Meanwhile, the suppression of the ECM degrading enzymes such as collagenase MMP presents a plausible approach to develop a strategy against OA [3]. In this study, we observed that miR-34a was markedly elevated in cartilage tissues of OA patients and strongly correlated with ECM degradation (MMP3, MMP13 and COL II)- and inflammation (iNOS, COX-2, TNF-α and IL-6)-related gene expression (Figure 1f). Therefore, we hypothesized that miR-34a plays a major role in ECM degradation and inflammatory response in OA.

Integrated bioinformatics prediction, GO enrichment analysis and KEGG pathway enrichment analysis revealed that MCL1 is a putative target of miR-34a in chondrocytes (Figure 2a-c). MCL1, an anti-apoptotic member of the BCL-2 protein family, is a main mediator of survival that suppresses intrinsic apoptosis or programmed cell death, and its expression is controlled by multiple mechanisms [19]. For instance, the 3ʹUTR of MCL1 has been indicated to be bound by different miRNAs, including miR-125b and miR-153-3p in different cell systems [20,21]. However, its direct binding relation with miR-34a (Figure 2d), to the best of our knowledge, has been rarely investigated before. Later, we sought to determine the upstream mechanism of miR-34a in OA. Interestingly, loss of DNMT1 lowered the methylation level of the miR-34a promoter and elevated the miR-34a expression in pancreatic cancer cells [22]. Therefore, we wonder whether the miR-34a expression was dependent on the regulation of DNMT in chondrocytes as well. As expected, the methylation of miR-34a promoter at −272/380 bp region was remarkably elevated in OA chondrocytes (Figure 3b), and DNMT3B protein was significantly enriched in the promoter region of miR-34a (Figure 3d). Moreover, a negative correlation between miR-34a and DNMT3B expression, and a positive correlation between MCL1 expression and DNMT3B expression were identified in cartilage tissues of rats with OA (Figure 3f).

To substantiate the regulatory function of DNMT3B/miR-34a/MCL1 axis, we delivered miR-34a inhibitor alone or silencing of DNMT3B and MCL1 into chondrocytes and rats with OA (Figure 4a). Kim *et al*. found that miR-34a was a negative regulator of chondrogenesis, particularly during the migration of chondroblasts [23]. Abouheif *et al*. reported that depletion of miR-34a significantly prevented downregulation of Col2a1 and upregulation of iNOS induced by IL-1b [24]. Moreover, inhibition of miR-34a prevented IL-1b-induced ECM degradation in nucleus pulposus [25]. While Cheleschi *et al*. established that miR-34a specific inhibitor curbed chondrocyte apoptosis induced by Visfatin [26]. Likewise, we observed that miR-34a inhibitor promoted chondrocyte proliferation (Figure 4), and repressed ECM degradation (Figure 5) and inflammatory responses (Figure 6). Izumi *et al*. found that the BBF2H7-ATF5-MCL1 pathway repressed endoplasmic reticulum stress-induced apoptosis in chondrocytes [27]. As regards to the function of DNMT3B, its knockdown led to decreased expression of the Col2a1 and increased expression of MMP13 [28]. More importantly, DNMT3B is essential for normal chondrocyte hypertrophic maturation and limb development, and loss of DNMT3B in chondrocytes delayed endochondral ossification and fracture repair [29,30]. In addition to the effects of silencing of DNMT3B and MCL1 in chondrocyte apoptosis (Figure 4) and ECM degradation (Figure 5), we also provided evidence corroborating the promoting effects of si-DNMT3B and si-MCL1 on OA *in vivo* (Figure 6).

Lastly, we conducted Western blot to attest the involvement of the PI3K/AKT signaling in the DNMT3B/miR-34a/MCL1 axis-mediated events. According to a recent review, the PI3K/AKT signaling is critical for normal metabolism of joint tissues, and is also involved in development of OA [31]. miR-34a inhibitor was found to expedite the pathway activity, which was reduced by DNMT3B or MCL1 knockdown. Consistently, miR-34a contributed to chondrocyte death and OA progression through modulation of the PI3K/AKT pathway [32]. Silencing of MCL1 promoted senescence and apoptosis in glioma cells by impairing the PI3K/AKT signaling pathway [33]. Moreover, miR-451a promoter methylation regulated by DNMT3B expedited the progression of bladder cancer via the PI3K/AKT axis [34]. All these findings were line with our observation that DNMT3B/miR-34a/MCL1 axis governs the OA progression via the PI3K/AKT signaling pathway (Figure 7a, b).

## Conclusion

**Figure 8.**
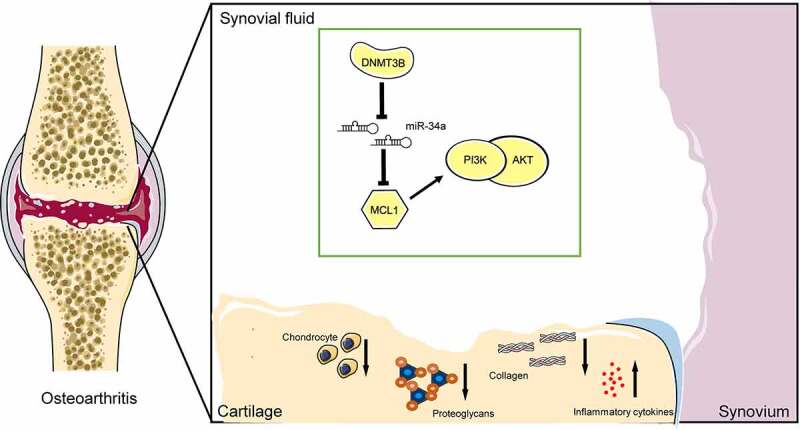
A model for the proposed role of miR-34a in OA. Inhibition of MCL1 expression by miR-34a impaired the PI3K/AKT pathway and promoted OA progression by inhibiting chondrocyte activity, promoting proteoglycan and collagen loss, and increasing inflammatory factor concentrations. DNMT3B, on the other hand, inhibits miR-34a via epigenetic suppression

Taken together, this body of work suggests that miR-34a upregulation, which was in close relation with DNMT3B loss, is tightly linked to ECM degradation and inflammatory response by binding to MCL1 and regulating the PI3K/AKT signaling in OA (Figure 8). These findings designate that miR-34a may be an attractive target for OA treatment. Nevertheless, further advanced studies are necessary to treat the cells with methylation inhibitors such as 5-AZA or (-)-epigallocatechin-3-gallate to confirm the upregulation of miR-34a and its associated effect.

## Data Availability

All the data generated or analyzed during this study are included in this published article.
